# Examining audience perceptions of sexist item songs: A contemporary analysis of bollywood

**DOI:** 10.1016/j.mex.2025.103214

**Published:** 2025-02-12

**Authors:** Forhana Choudhury, Swati Sharma

**Affiliations:** School of Social Sciences and Languages, Vellore Institute of Technology, Vandalur – Kelambakkam Road, Chennai 600127, Tamil Nadu, India

**Keywords:** Sexist songs, Bollywood, Male gaze, Reinforcement theory, Cognitive Dissonance, Mixed Methods Approach

## Abstract

Bollywood, better known as the Hindi cinema industry, is globally renowned for its entertainment, yet it grapples with a persistent issue - the prevalence of sexist item songs. These dance sequences often objectify female actors, but prevalent research has predominantly focused on their consequences rather than the reasons behind their continued creation and acceptance.

The paper traces the historical evolution of item songs and their socio-cultural root causes responsible for production and distribution. A mixed method approach was employed, data were collected from 310 working professionals using a convenience sampling technique and a thorough review of 62 articles from databases like Jstor, ProQuest, Google Scholar, and Springer. By employing a mixed method approach, this research statistically analyses the critical socio-cultural factors contributing to sexist song production, the societal impact of such songs, and the root causes of their depiction.

• The approach explores how Bollywood reinforces gender norms through rewards and punishments, perpetuating the conformity of women to stereotypical roles.
• Additionally, it investigates the Male Gaze theory in the context of Bollywood's portrayal of women as objects of desire.
• It applies Cognitive Dissonance to understand the audience's resistance to change despite awareness of the harm caused by sexist songs.

## Graphical abstract

**Figure:**
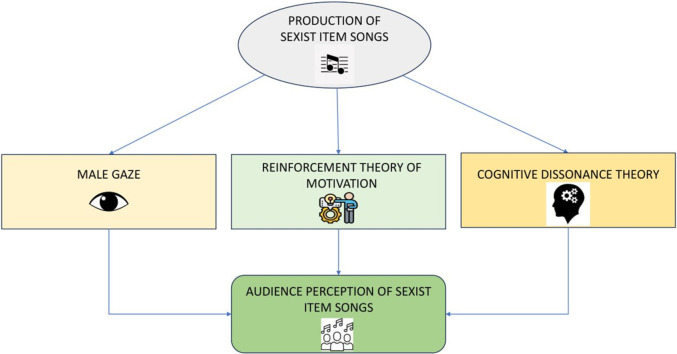

Specifications table

*This table provides general information on your method.*

**Table:** 

Subject area:	Psychology
More specific subject area:	Gender Representation in Media and its Effects on Audiences
Name of your method:	Mixed Methods Approach
Name and reference of original method:	Creswell, J. W. (2014). Research Design: Qualitative, Quantitative, and Mixed Methods Approaches.
Resource availability:	Data collection tools (surveys), databases for literature review (JSTOR, ProQuest, Google Scholar, Springer).

## Background

The motivation behind employing a mixed-methods approach in this study stems from the necessity to comprehensively explore the complex socio-cultural dynamics surrounding the production and acceptance of sexist item songs in Bollywood. This research places itself at the intersection of media studies, gender studies, and cultural sociology. Bollywood, as a significant cultural identifier within Indian society and a global entertainment force, warrants an analysis that critiques both its artistic expressions and its implications on social norms. By bringing together qualitative and quantitative methods, this study seeks to bridge the gap between theoretical frameworks, such as the Male Gaze theory and Cognitive Dissonance, and empirical data gathered from industry professionals and scholarly literature. By employing qualitative measures, such as interviews and thematic analysis, the research identifies the cultural narratives and stereotypes that have been amplified over decades.

The mixed-methods approach allows for a robust statistical analysis of data collected from 310 working professionals within the industry, which supports qualitative insights from a review of 62 scholarly articles. This dual analysis facilitates a more inclusive interpretation of the findings, uncovering the socio-cultural factors that contribute to the prevalence of sexist songs. It also enables the examination of audience perceptions and their varied responses to these portrayals, highlighting the resistance to change despite an awareness of the harmful implications, a phenomenon explained through the lens of Cognitive Dissonance. Understanding the root causes and societal impacts of item songs is crucial in understanding gender representation in media and advocating for a positive change within the film industry.

## Method details

### Utility and popularity of item songs

Bollywood, popularly known as Hindi cinema across the world, is identified as the hub of entertainment films made for the Indian audience. Item songs are defined as dance numbers placed exclusively in Indian films with or without context, focusing solely on the objectification of the female actor. Item songs in Bollywood are the hub of sexist songs today. The reasons as to why these songs still continue to be created and disseminated within the masses is a question frequently asked within academia. Perhaps the existing research focuses much on the after-effects of these sexist item songs on women rather than addressing the root causes/factors of this issue.

The prevalence of item songs in the present decade would be a result of all the developments in Indian cinema in the past: from women initially being divided into the binaries of the virgin and the vamp [1]. It was only later in the 1990s that the term “item girl” came to be exclusively known and addressed, from a dance number that Shilpa Shetty (a popular Indian actress) had performed, completely unaware of the consequences of that dance number on the Indian audience today.

Since then, the utility and popularity of item songs have been shifting, even now, when item songs are not merely a luxury added to the film, but a complete necessity to keep the film from falling apart [2]. The item songs today, carry within themselves the power to either make a movie or break a movie. Consequently, the actresses or rather dancers who are assigned these songs carry heavy demand and are acknowledged by the entire Indian population, sometimes even overshadowing the popularity of the female lead in the same film [3].

The consequences of item songs have been discussed in abundance so far but the major socio-cultural factors responsible for this particular pattern of creation and dissemination of sexist songs is an area yet to be explored in detail [[4], [5], [6]]. Tracing the major socio-cultural factors would thus, help us understand the exact reasons as to why these sexist songs still continue to be created and accepted within the Indian society. To understand the same, it would be imperative for us to explore the current trends of item songs in the country, and how they enable a deep conditioning of the acceptance of sexist item songs within the Hindi film industry, better known as Bollywood. This paper, therefore, attempts to examine in detail, the exact hindrances/causes that aid in promoting the conventional pattern of sexist item songs in Bollywood.

### Male gaze in bollywood

To begin with, the Male Gaze perspective has not only prevailed within Bollywood movies but also permeated to the Bollywood songs produced for these movies [7]. However, the discussions around Male Gaze in Bollywood have been limited to the ramifications that this gaze would have on the population, particularly on women. Indian cinema fundamentally focuses on the conventional pattern of projecting the man as the lead protagonist and the woman as the supporting character, or an object to simply accentuate the storyline. From this perspective, most of the films are designed to portray the woman as an object of desire or tool to advance the plot. Needless to say, the songs that are produced would also carry the same Male Gaze perspective [8,9].

### Reinforcement theory of motivation

Furthermore, the practice of rewards and punishments has been reiterated by Bollywood across decades of filmmaking. It has been observed that women who complied with stereotypical gender norms avoided punishment or received fewer punishments as compared to women who did not adhere to the same [10]. This concept of rewards is also endorsed by Bollywood in its songs to incorporate the notion that women who complied with and accepted the sexist norms set by society received appreciation or praise in comparison to other women. This is attested through the Reinforcement Theory [11], which describes how viewers seem to identify more with a character conforming to their belief systems: therefore, they conform to the existing gender prejudices. While women conform to the idea of evading punishment (as expressed in these songs), men also conform to the idea of rewards as exhibited through the same songs, wherein the male actors are rewarded for exhibiting sexist behaviour towards their female counterparts.

### Cognitive dissonance theory

Cognitive Dissonance refers to the breaking away from a conventional way of thinking in an individual. Leon Festinger, a psychologist, introduced Cognitive Dissonance in 1957 as the condition when people's thoughts and feelings are not in accordance with their behaviour, which results in psychological discomfort of the mind and the body. This may cause distress in that individual or further stress [12]. As humans, we are designed to follow a set course, and dissociating from that course often ends up being unpleasant and discomforting for us. Cognitive Dissonance, therefore, could be applied in the areas of sexist music as well as in the production of films in Bollywood. Within the framework of Music Sociology, we find that songs contribute abundantly to the formation of social realities [13]. The consumption of sexist songs has been so internalized by the audiences that introducing them to something different or unfamiliar or (non-sexist) could result in distress to the audience who might not associate with the protagonists or the supporting actresses [14].

Three critical aspects regarding sexist music produced in Bollywood are examined here. Firstly, Laura Mulvey's theory examines how these songs are fashioned through the Male Gaze and subsequently become highly problematic to the masses. Secondly, the production of these sexist songs is reinforced by the audiences through the Reinforcement Theory of Motivation, in which, the masses identify with the (conventional sexism) in item songs and allow it to be reproduced and (reinforced) over and over again. Finally, the Cognitive Dissonance Theory would help identify how it is extremely difficult for the common masses to do away with sexist songs, even if they are slightly aware of how perilous it is to society, due to continuous reinforcement and normalization of sexist ideologies.

## Method details

### Nature of study

The nature of study for this paper is a mixed method research (combination of quantitative and qualitative methods), encompassing primary data collection using a survey-administered questionnaire and literature review based on 62 articles. The items in the questionnaire were measured using a “5-point Likert scale” and “binary questions”. Using convenience sampling (a non-probability sampling approach), 350 questionnaires were distributed among the participants, and recorded 310 valid responses at a response rate of 88.6 %. In terms of the qualitative approach, this research used these articles to elucidate on the most problematic areas regarding the topic, stating the critical socio-cultural factors or root causes that contribute to the production and dissemination of these sexist songs. Furthermore, they also discuss the multitude of ways in which these songs affect the psyche of the Indian masses and influence their behaviours as well.

### Data base

Electronic databases such as Jstor, ProQuest, Google Scholar, Springer, among others, were used to derive appropriate searches relating to the topic. The searches were carried out to scan articles pertaining to the keywords- Bollywood, sexist lyrics, item songs, Male Gaze, Capitalism, Purgation effects, etc. Relevant articles published from 1990 to 2022 were selected for the study.

Articles focusing on the prime factors such as discussion on the objectification of women based on Bollywood music, the problematic portrayal of women in sexist songs and Bollywood songs in particular, how these visual representations reflect on the Indian audiences and most importantly, the root causes of these sexist depictions in songs are included in the study.

### Qualitative analysis

So far, it is observed that there is a conventional pattern followed when it comes to the production and dissemination of sexist songs in Bollywood. This conventional pattern allows neither the producer nor the Indian consumer to shift away from the conventional production and consumption of these sexist songs. It is only the repercussions that have been traced and discussed so far in detail. This section, therefore, has focused on what exactly are the socio-cultural factors/difficulties in breaking the conventional pattern of sexist item songs in Bollywood. Three major divisions have been identified and discussed as follows.

### Masculine control and objectification of women

#### Influence of male lyricists

The first factor identified under the Masculine Control and Objectification of Women is derived from male lyricists highly influencing the production of item songs. The most integral aspect of a song could be identified as the lyrics of the song, and majorly influences the direction towards which the song is headed. Subsequently, sexist songs are designed based on the lyrics titillating not just the audience, but also the male and female actors, the director as well as other viewers [15]. The reason as to why a majority of these songs fall into the purview of sexism is because of the male gaze that is deeply ingrained within the song, mostly offered by male lyricists and writers [16].

The availability as well as professional practices of female lyricists remain extremely handful in Bollywood, and even with female lyricists entering the business, the male perspective is always implemented in their writing for the consumption of a patriarchal Indian audience. It could be deduced that the male lyricists are, therefore, projecting their biases whether consciously or subconsciously. These biases portray the conventional pattern of the distinctive patriarchal ideology of a dominant, controlling male as juxtaposed to a powerless, submissive female [17,18].This pattern is heightened with the performance of the female actress endorsing these lyrics and normalizing them to a great extent, so much so that it is made to believe that women in fact, enjoy being described in a sexist manner through these lyrics [19,20].

This factor is highlighted under the male perspective due to the large influence of male lyricists mostly, who endorse the patriarchal perspective of viewing women in two distinct binaries in their songs. The songs, therefore, are also written in the same manner, with derogatory descriptions of women in either of the binaries (as the Madonna or the Whore). Bollywood item numbers, which are standalone dance sequences often featuring scantily clad women, are a common source of the Madonna-Whore complex. These sequences tend to objectify female dancers, depicting them as objects of desire for the male gaze. While these numbers are meant for entertainment, they can reinforce the idea that women exist primarily for male pleasure. This reason hence, reinforces the emergence and participation of more male lyricists within the music industry which leads to more sexist item songs being created in Bollywood in the present scenario.

#### Bollywood's control over female sexuality

A crucial element that highlights the Masculine Control is Bollywood's control over female sexuality. It is an established norm that the directors expect the audience, both within the film and outside, to be male viewers. The implications of this norm are that the male actors are the designers of this objectification of women's bodies on screen and propagate it to their male viewers as well. They further direct the internalized sexism within the masses and normalize it to the extent that objectification of women's bodies is practiced in society without any questioning [12,21].

The appreciation for the “female body” that is glamorized in the songs by the male lead, is not simply limited to the film itself, it then surpasses the screen and onto the behaviours of men in society who accept this objectification of the female body [22]. The connotations associated with the objectification of the female body are normalized as praises to the woman, which in reality turns out to be nothing less than verbal harassment.

This perspective speaks for itself, in ways that Bollywood's notion of sexuality is largely controlled by a group of powerful men who decide what is to be censored and what is acceptable to society [23,24]. Between these blurred lines of what is acceptable and what is not, most often covert and overt forms of sexism are left out by the Censor Board and the authorities in Bollywood. Female sexuality, as with all cinemas over the world, is highly controlled by the Male Gaze. The levels of nudity allowed and morals attached to a woman's clothing are thus defined by the same [25].

#### Socio-Political power, capitalism and purgation effects

Socio-Political power, Capitalism, and Purgation effects are all inter-related and affect each other respectively when it comes to the production of these sexist songs. The Socio-Political power is said to be situated under the male perspective as it is extremely controlled by patriarchy and to the undue advantage of women [26]. The Socio-Political power structure of a country majorly contributes to the kind of music or songs that is produced or disseminated to the masses. In the past, certain songs or films have been banned from the public purview due to the attempt of breaking away from the conventional pattern of sexist songs or movies. This gave rise to nationwide unrest and even violence in some areas [27,28].

With the onset of Capitalism, music found its own way to reach to Indian audiences, and the process of money-making only aided in the distribution of music that appealed to the taste of the male audiences [15]. This method was largely followed from the West and particularly from Hollywood, trying to integrate sexist attitudes in their music without the concern for violence against women [29]. While Capitalism has always worked hand in hand with patriarchy and its consequences mostly favour men (while at the expense of women's rights and their overall health), it could be said that women too are a part of the larger purview of Capitalism and contribute significantly towards its growth.

The Purgation effect, however, is completely different from the Capitalistic view, as it focuses more on the audience's personal consciousness. This could be understood from the reason as to why the masses watch a song in the first place [30]. They are able to relate to the protagonist in a certain manner, and therefore, the song provides a feeling of Purgation which leads to a form of mental relaxation in people [31,32]. This is related to the Cognitive Dissonance Theory in the way that people find comfort in what they identify as familiar and a distaste for what they are unaware of. Due to this Purgation effect, most of the songs are created in the conventional pattern of sexist music in Bollywood [33].

### Shouldering the burden: needs of women

#### Uncertainty of the livelihood of female actors

Accordingly, the issues that are stimulated by women include the livelihood of female actors, the division of female dancers into three categories, and celebrity endorsement and female career [34,35]. It is apparent that the livelihood of the female actors depends on these sexist item numbers as they are currently the better option for dancers struggling to acquire a stable job, or dancers working in bars or hotels or in local shows [36]. This is covered under the theory of Cinesexuality [37,38], which discusses the underlying reasons as to why item numbers or sexist songs cannot be done away with, even when we are aware of their repercussions on the common milieu.

This factor is perhaps one of the fundamental root causes for the abundant production and dissemination of sexist songs in Bollywood, and the industry feeds off of the lack of proper jobs for the middle classes, especially for female actors. The unavailability of regular incomes and stable jobs in acting, or dancing for that matter, pushes a majority of the women to incorporate the conventional pattern of sexist songs within their routine and normalize objectifying their own bodies on camera for the male viewers [39,40].

When it comes to livelihood, sexualization or exploitation of bodies is brushed aside to provide a stable income source to the family first. The film industry thus, propagates this heinous cycle of exploiting under-paid artists and regulates the production of item songs by employing these artists to contribute to the same [41]. This is listed as yet another crucial factor as to why the middle-class audience especially, is unable to break away from the conventional pattern of sexist music in Bollywood [42].

#### Division of the female character into three categories

An inherent factor that is associated with female actors is the distinct division of women into three categories – a) the vamp b) the dancer by profession c) the heroine's noble sacrifice. Bollywood did have to come up with certain diversions for women characters to be allowed to dance in front of a male audience [43]. It allowed women to perform dance numbers against the prescribed Indian moral codes. This in itself, has been internalized now and accepted by the Indian audience and a dance number is much revered and enjoyed by the masses today, so much so that these performances are hosted in celebrity dance shows and award functions.

Consequently, the first division was that of the “vamp”, which was carefully designed back in the 70 s for the pleasure of the male audience. This had come up from the cabaret dancers, but the vamp included a more radical set of characteristic traits. This was the woman who was immoral and seductive and was the polar opposite of the virgin heroine [7]. She was associated with negative roles and would also be a tool to carry forward the storyline for the male protagonist. However, her dance numbers were exactly what set her apart from the rest of the actors. It allowed her a means of freedom along with the burden of the promiscuous woman [44].

The second was the dancer by profession. This was the woman who was at the crossroads of morality and could easily carry out an overtly sexualized dance number, but of course, for the right moral reasons [8,45]. This could be for a charitable cause such as financial insecurity for the heroine, as the actress, Madhuri Dixit would perform similar characters during the late 90′s in her item numbers. Her dance would be justified as the need for her profession, and without the onus of placing her within the binaries of a “good” or “bad” woman [[46], [47], [48]].

The third was the heroine's noble sacrifice. This would be self-explanatory as “the heroine performing a dance number in order to save the male protagonist” [49]. These justifications of dance numbers by the lead actresses and the classifications of women into three sexist groups, thus further strengthened the need for item numbers in Bollywood, consequently making it even more difficult to break away from the conventional pattern of sexist music.

These three categories significantly arise from the need to incorporate songs without any context to the movie, but allow the viewers to watch the movie, especially for the song [50,51]. These divisions are also created in order to further the already-used plotline in the movie [31,52]. As a result of these divisions in item numbers, women are actually demarcated as the “good, virtuous or the pious woman” or the “vamp, promiscuous woman” in real life as well. This also falls under the larger purview of the Madonna-Whore Dichotomy, wherein women are divided and distinguished based on characteristics such as “virtue” or “promiscuity”. The prejudice thus held, against women for behaving a certain way or breaking away from the conventional pattern of sexism allotted to the society, is highlighted clearly through this issue.

#### Celebrity endorsement and female career

Celebrity endorsement and female career are also root issues that influence female psychology in Bollywood. This is one factor where women are consciously contributing to the already existing internalized sexism within the industry and further propagating it. Celebrities most often endorse sexist item numbers with the justification that they are catchy and played in most weddings or bars, discos, and other important events [53]. The actors agree upon performing these songs, as this is quick work with a large amount received by the end of the song [31,54]. Furthermore, once the song is popular enough, the actors or actresses are invited to perform the same at weddings, award ceremonies, and events, which leads to more “quick money”. The most imperative reason as to why female actresses today agree to perform these item songs is because the item girl overshadows the female lead actress in that movie simply by performing one song [24].

Since it is already known that the “shelf life” of an actress is quite limited in Bollywood, actresses make use of item songs as much as possible to garner the attention and fame of their audiences as soon as possible. Without item songs, it could be stated that their career would end within a decade or even less. This is one factor that seems to be refutable, provided that female actresses take the initiative of saying no to item songs while understanding the repercussions that they cause to women in real life [55].

### The aftermath of technology

#### The digitization of item songs

The digitalization of item songs would mean the assimilation of the item girls’ performances into the larger arena of Capitalism that has been propelled forward today by technological advancements in the entertainment business [56]. With the advent of the Internet and multiple streaming devices today, the dissemination of sexist songs and an ingrained sexist ideology travel much faster and reach more audiences than it did in the last two decades.

Multiple gadgets such as iPhones, iPods, etc. allow the easy transfer of music within a short period of time across a larger space. This entirely transformed the experience of the consumers: music was now decentralized and easily transported from one platform to another [44,57]. The authority that Indian consumers now felt over music was manifold, from being simply consumers at one point; they now viewed themselves as the bearers of music [36,58]. Within this mayhem, item songs found a new portal to move ahead swiftly, to circulate themselves even more freely than ever before, into households, and into the psyche of the masses.

The central premise of item songs was now placed as the “Saviour” of the film, and the latter depended completely on its survival at the box office. It is in this way that technology accentuated the arrival of item songs and placed it as a household commodity within the Indian diaspora [59]. Digitalization is governed by male preferences (through Capitalism) and influences the way women accept and share this form of conventional representation. The Reinforcement Theory of Motivation as discussed in the above sections, could also be identified here, as it explains how item songs are continuously being made in the present era as the masses are able to relate and conform to the sexist beliefs displayed in these songs, thus creating a space for its commercialization in Bollywood.

## Statistical analysis (Quantitative)

### Respondents’ profile

The demographic characteristics of the participants are demonstrated in Table 1. The population of this study involves 310 working professionals who provided valid responses. In terms of gender, the measures of both male (51 %) and female respondents (49 %) are approximately equal. On measuring the age category of participants, 34 % of the population are aged between 18 and 25 years, 30 % of participants are aged between 26 and 35 years, 24 % of respondents belong to the age group “36 to 45 years”, 7 % of participants are aged between 46 and 55 years, and only 5 % of the population belong to the age category “56 years and above”. As demonstrated in Table 1, 22 %% of the study's population have a “Diploma”, 40 % of respondents are undergraduates, 29 % of them are postgraduates, 6 % of participants are Doctorates, and 3 % of respondents are “others”. Moreover, data collection of this study has been conducted in four districts in Tamil Nadu, where 37 % of the population is from Chennai, 30 % of the participants have their location in Chengalpet district, 22 % of the population is from Kancheepuram district, and 11 % of the population is from Thiruvallur district.

**Table 1:** Demographic Characteristics of Respondents.

Measures	Items	Frequency (N = 310)	Percentage
***Gender***			
	Male	158	51 %
	Female	152	49 %
***Age***			
	18 to 25 years	106	34 %
	26 to 35 years	094	30 %
	36 to 45 years	073	24 %
	46 to 55 years	021	07 %
	56 years and above	016	05 %
***Education***			
	Diploma	067	22 %
	Undergraduate	125	40 %
	Postgraduate	089	29 %
	Doctorate	020	06 %
	Others	009	03 %
***Employment Status***			
	Private Employee	156	50 %
	Govt. Employee	078	25 %
	Self-employed	054	17 %
	Others	022	08 %
***Locality***			
	Chennai	114	37 %
	Chengalpet	094	30 %
	Kancheepuram	068	22 %
	Thiruvallur	034	11 %

### Descriptive statistics

The statistical analysis of the quantitative data was carried out using SPSS-AMOS. The respondents were asked to answer the questions using the questionnaire items with the objective of measuring audience perceptions of sexist item songs of Bollywood. Descriptive statistics was conducted to ensure the relationship between the variables. Table 2 represents the results of descriptive statistics, concerning the perceptions of the audience (both male and female) toward the sexist item songs.

**Table 2:** Audience perceptions of sexist item songs.

Measures	Male (158)	Female (152)	p-value	Outcomes
Male lyricists’ influence	86.70 %	79.40 %	0.000^⁎⁎⁎^	Significant relationship
Control on female sexuality	82.10 %	74.60 %	0.000^⁎⁎⁎^	Significant relationship
SCP	79.60 %	84.90 %	0.000^⁎⁎⁎^	Significant relationship
Livelihood uncertainty of female actors	85.40 %	78.40 %	0.000^⁎⁎⁎^	Significant relationship
Celebrity endorsement	87.80 %	84.50 %	0.000^⁎⁎⁎^	Significant relationship
Digitization of item songs	84.20 %	81.60 %	0.000^⁎⁎⁎^	Significant relationship

*(Note:* SCP – Socio-political, Capitalism, Purgation effects; *p < 0.05, **p < 0.01, ***p < 0.001).

As demonstrated in Table 2, all measurement items of this study ensured a statistically significant relationship with the perceptions of the audience toward sexist item songs (since p-value = 0.000 < 0.05).

### Reliability and validity test

This research used the CFA test (Confirmatory Factor Analysis) to assess the reliability and validity of the study's model and questionnaire instrument as illustrated in Table 3.

**Table 3:** Reliability and validity analysis.

Measures	Factor loadings	Cronbach's alpha	Composite Reliability	Average Variance Extracted	p-value
Male lyricists’ influence	.83	.91	.90	0.59	0.000
Control on female sexuality	.81	.87	.92	0.57	0.000
SCP	.86	.84	.89	0.58	0.000
Livelihood uncertainty of female actors	.79	.78	.81	0.56	0.000
Celebrity endorsement	.84	.83	.80	0.59	0.000
Digitization of item songs	.87	.89	.87	0.51	0.000

*Note:* SCP – Socio-political, Capitalism, Purgation effects.

Table 3 illustrates the measure of reliability (Cronbach's Alpha), convergent validity (AVE), factor loadings, and composite reliability of the items. The CFA test results highlight that “χ2 (df) = 17.84, *p* < 0.001, goodness of fit (GFI) = 0.99, adjusted goodness of fit (AGFI) = 0.98, and root mean square error of approximation (RMSEA) = 0.07″ are found to acceptable range, confirms a good model fit [60]. Moreover, the measurement of AVE for the items indicates a stronger convergent validity, since the values are found to be ahead of the threshold value “0.5″. On the other hand, the reliability (Cronbach's Alpha) test results of the items are greater than the standard value “0.7″, indicating the study's instrument is reliable and achieved good internal consistency. However, “the composite reliability results of the six items are found to be higher than the standard value 0.6, indicating the convergent validity of the constructs are acceptable” [61]. Further, the factor loadings of each item are higher than the threshold value “0.7″, found to be acceptable. Finally, the discriminant validity (as given in Table 4) was examined using “HTMT (heterotrait-monotrait) ratio of correlations” [62].

**Table 4:** Heterotrait-Monotrait Ratio.

Constructs	1	2	3	4	5	6
Male lyricists’ influence	–					
Control on female sexuality	.75	–				
SCP	.73	.81	–			
Livelihood uncertainty of female actors	.66	.77	.84	–		
Celebrity endorsement	.48	.58	.59	.64	–	
Digitization of item songs	.51	.56	.64	.68	.71	–

*Note:* SCP – Socio-political, Capitalism, Purgation effects.

The discriminant validity of the study can be examined through the “HTMT ratio of correlations” as given in Table 4. Since the correlated values of HTMT ratios for each item fall within the threshold value “0.85″, it indicates the attainment of a good discriminant validity.

Table 5 demonstrates the measure of “correlation, mean and standard deviation”. The correlation value of each item indicates the existence of positive correlations, thus highlighting that sexist item songs of Bollywood have a statistically significant positive relationship with the perceptions of the audience.

**Table 5:** Correlation, SD and Mean.

Constructs	1	2	3	4	5	6	Mean	SD
Male Lyricists’ influence	1	063^⁎⁎⁎^	.52^⁎⁎⁎^	.39^⁎⁎⁎^	.32^⁎⁎⁎^	.45^⁎⁎⁎^	4.01	1.09
Control on female sexuality	.45^⁎⁎⁎^	1	.59^⁎⁎⁎^	.60^⁎⁎⁎^	.47^⁎⁎⁎^	.54^⁎⁎⁎^	4.29	1.29
SCP	.27^⁎⁎⁎^	.72^⁎⁎⁎^	1	.69^⁎⁎⁎^	.41^⁎⁎⁎^	.39^⁎⁎⁎^	3.81	1.32
Livelihood uncertainty of female actors	.80^⁎⁎⁎^	.75^⁎⁎⁎^	.57^⁎⁎⁎^	1	.62^⁎⁎⁎^	.59^⁎⁎⁎^	4.11	1.26
Celebrity endorsements	.44^⁎⁎⁎^	.54^⁎⁎⁎^	.43^⁎⁎⁎^	.61^⁎⁎⁎^	1	.67^⁎⁎⁎^	3.94	1.56
Digitization of item songs	.45^⁎⁎⁎^	.61^⁎⁎⁎^	.58^⁎⁎⁎^	.67^⁎⁎⁎^	.69^⁎⁎⁎^	1	4.31	1.57

*Note:* SCP – Socio-political, Capitalism, Purgation effects.

## Method validation

Since this study adopted a mixed-method approach, first, the qualitative segment discusses that the Male Gaze holds a substantial presence within the Bollywood music industry, where it profoundly influences the creation and depiction of female characters in Hindi songs and films. This gaze reduces women to objects of desire, often lacking agency or depth, reflecting a pervasive issue not exclusive to Bollywood but prevalent in global media. The interplay of the Male Gaze with capitalism and patriarchy emerges as a central concern, as Bollywood's patriarchal perspective, influenced by Western narratives, particularly Hollywood, underpins the industry's gender dynamics. This perspective, while instrumental in fostering the industry's commercial success, disproportionately impacts women and minority groups.

Moreover, female celebrities, in their pursuit of artistic expression and economic stability, also contribute to the perpetuation of patriarchal values, navigating a complex environment with limited options for non-sexist content. Furthermore, the normalization of sexist songs among female audiences, driven by the Purgation effect, sustains the acceptance and celebration of such content. This normalization enforces the division of women into binary/dichotomous roles - the virtuous Madonna and the objectified Whore - erasing their multifaceted identities and curbing their individuality.

As portrayed in these sexist songs, the women in real life too, try to remain within the category of the Madonna, and in case a woman abstains or goes away from the category, she is termed as the “Whore” who does not adhere to the conventional sexist rules stated by the patriarchal society. Women here, are strong propagators as well as practitioners of patriarchy and influence this division so much, that while sexist item songs are enjoyed by many, women in real life would not want to fit into the role of the item girl. Some Bollywood songs may depict male characters as having the freedom to pursue sexual relationships without judgment, while female characters are portrayed as needing to maintain their purity and chastity. This reinforces traditional gender roles and contributes to the Madonna-Whore complex.

Authenticity in the portrayal of women is frequently missing in Bollywood. The characters women play often lack depth, real-world complexity, and the ability to represent the diverse experiences of actual women. This absence of authenticity can contribute to a skewed understanding of women's lives and struggles, perpetuating misconceptions and stereotypes. Sexist songs are a significant concern as they contribute to the perpetuation of harmful stereotypes. While OTT platforms today have made significant strides in promoting female-oriented filmmaking and familiarizing the audience with the female gaze, with films such as *Laapataa Ladies* (2023), *Gangubai Kathiawadi* (2022), *Margarita with a Straw* (2014), there still remains a considerable gap in mainstream Indian cinema's approach in dismantling the conventional pattern of film making.

Second, the quantitative analysis results of this study symbolize that perceptions of the audience have a positive relationship with the measurement items, according to the descriptive statistics results (see Table 2). Moreover, the correlation test results suggest that there is a strong positive correlation between the questionnaire items and audience perceptions. Table 5 highlights that each item has a positive correlation and a statistically significant relationship between the items and audience perceptions. However, the mean and standard deviation values for each item were found to be acceptable. For the item “male lyricists’ influence” the mean value (mean = 4.02) and standard deviation (SD = 1.09), for “control on female sexuality”, mean value = 4.29 and SD = 2.29, the measure of “SCP” highlights (mean = 3.81, SD = 1.32), for “Livelihood uncertainty of female actors”, the mean value measures (mean = 4.11, SD = 1.26), the mean value and standard deviation of celebrity endorsement accounts for (mean = 3.94, SD = 1.56), for “Digitization of item songs” mean value = 4.31, standard deviation = 1.57). Furthermore, correlation analysis of all the items ensured a positive correlation and a strong relationship between audience perception and portrayal of women in sexist item songs. Third, based on the qualitative and quantitative analysis, the test results highlight there are positive and similar results. This confirms that the mixed-method approach employed in this study is reliable and consistent.

## Conclusion

Within academia, the debates and discussions around sexist songs in Bollywood have always focused on the effects of these songs on the masses, especially on women. There has been abundant existing research supporting the same. However, the root causes of the production and distribution of these sexist songs have never been explored in detail so far. The perpetuation of harmful stereotypes, the restriction of women's sexuality, and the dual role of women as both perpetrators and victims of their own actions exacerbate the complexity of their roles in society. To address these issues, increased awareness, a diversification of creative roles, and a re-evaluation of deeply ingrained gender norms remain highly essential.

## Limitations

Not Applicable.

## Ethics statement

None.

## CRediT authorship contribution statement

**Forhana Choudhury:** Methodology, Conceptualization, Writing – review & editing. **Swati Sharma:** Writing – review & editing.

## Declaration of competing interest

The authors declare that they have no known competing financial interests or personal relationships that could have appeared to influence the work reported in this paper.

## Data Availability

Data will be made available on request.
